# VSIG4 Induces Epithelial-Mesenchymal Transition of Renal Tubular Cells under High-Glucose Conditions

**DOI:** 10.3390/life10120354

**Published:** 2020-12-17

**Authors:** Eun-Yeung Gong, Hyung Ah Jo, Sang Hyun Park, Dae Ryong Cha, Dae Young Hur, Sang Youb Han

**Affiliations:** 1 Department of Anatomy and Tumor Immunology, Inje University College of Medicine, 75 Bokji-ro, Busanjin-gu, Busan 47392, Korea; eunyeunggong@gmail.com; 2 Department of Internal Medicine, Inje University College of Medicine, Ilsan-Paik Hospital, Joowha-ro 170, IlsanSeo-gu, Goyang, Gyeonggi 10380, Korea; hyunga@paik.ac.kr; 3 Department of Urology, Inje University College of Medicine, Haeundae Paik Hospital, Haeundae-ro 875, Haeundae-gu, Busan 48108, Korea; urosh@hanmail.net; 4 Department of Internal Medicine, Korea University Ansan-Hospital, Kojan-Dong 516, Ansan, Kyungki-Do 15355, Korea; cdragn@unitel.co.kr

**Keywords:** V-set immunoglobulin-domain containing 4 protein, diabetic nephropathies, kidney, fibrosis, transforming growth factor beta

## Abstract

High glucose-mediated tubular injury contributes to the development and progression of diabetic nephropathy through renal tubulointerstitial fibrosis. V-set immunoglobulin-domain-containing 4 (VSIG4), a B7 family-related protein, is a complement receptor. Although the role of epithelial–mesenchymal transition (EMT) has been reported in several diseases, little is known about its relationship with VSIG4 under diabetic conditions. This study aimed to investigate the role of VSIG4 in human tubule cells stimulated by high glucose (HG, 55 mM). HG upregulated both mRNA and protein levels of VSIG4 in proximal tubule cells (HK-2 cells) and Madin Darby Canine Kidney cells. These upregulations were accompanied by increased expression of mesenchymal markers such as fibronectin, N-cadherin, matrix metalloproteinase 9, and vimentin, and by decreased expression of the epithelial marker, E-cadherin. The siRNA-mediated inhibition of VSIG4 in HK-2 cells restored the dysregulation of EMT in cells. Interestingly, VSIG4 inhibition did not affect the expression of transforming growth factor (TGF)-β, whereas inhibition of TGF-β reduced VSIG4 expression, subsequently suppressing fibrosis markers. These findings suggest that VSIG4 plays an important role in mediating renal tubular EMT through the downstream action of HG-induced TGF-β activation.

## 1. Introduction

The phenotypic conversion of kidney cells after injurious stimuli such as high glucose (HG) has a crucial role in the progression of diabetic nephropathy [1,2]. Accumulating evidence indicates that epithelial–mesenchymal transition (EMT) of the kidney cells, wherein the cells lose their epithelial features and gain mesenchymal phenotype, is one of the most prominent findings in diabetic nephropathy [1]. Given the pathogenetic role of EMT in the development of renal tissue injury in diabetic nephropathy, it is essential to elucidate the downstream mediator that leads to kidney fibrosis in diabetic nephropathy to be able to identify therapeutic targets [3,4].

V-set immunoglobulin-domain-containing 4 (VSIG4) is a B7 family-related protein that acts as a receptor for complement C3. Although its function is not yet understood, it has been reported to regulate T cell proliferation and activation [5,6]. VSIG4 is also overexpressed in various malignant tumors, including non-small cell lung cancer and glioblastoma, and plays a potential oncogenic role by regulating T cell proliferation, migration, and invasion [7,8].

Recently, VSIG4-related EMT was reported in renal cells as well as cancer metastasis [8,9]. Moreover, VSIG4 is upregulated in diabetic kidney disease, as indicated by bioinformatics analysis [10]. However, the precise role of VSIG4 in the diabetic milieu is still unclear. Considering role of transforming growth factor (TGF)-β on EMT in renal tubular cells, VSIG4 would be related to the TGF-β in the process of EMT. Accordingly, in the present study, we aimed to elucidate the role of VSIG4 in EMT through TGF-β signaling involved in kidney fibrosis under HG conditions.

## 2. Results

### 2.1. VSIG4 Upregulation by HG Stimulation in Renal Tubular Epithelial HK-2 Cells

**Figure 1:**
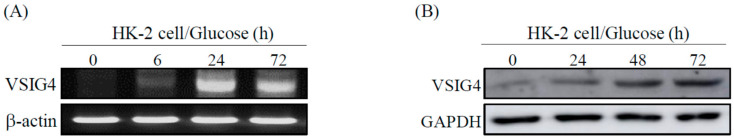
Upregulation of V-set immunoglobulin-domain-containing 4 (VSIG4) induced by HG condition in human proximal tubular epithelial cells (HK-2 cells). (**A**) HK-2 cells were incubated with HG for 6 h, 24 h, and 72 h. VSIG4 expression was determined by RT-PCR. (**B**) HK-2 cells were incubated with HG for 24 h, 48 h, and 72 h, and then subjected to Western blot analysis using anti-VSIG4 antibody. HG, high glucose.

To determine the association of VSIG4 with high glucose (HG)-induced injury, we investigated VSIG4 expression under HG conditions in human proximal tubular epithelial cells (HK-2 cells). Both the mRNA and protein expression of VSIG4 were dramatically increased in the HK-2 cells under HG condition in a time-dependent manner (Figure 1A,B).

### 2.2. EMT by VSIG4 Upregulation in Renal Tubular Cells Stimulated by HG

**Figure 2:**
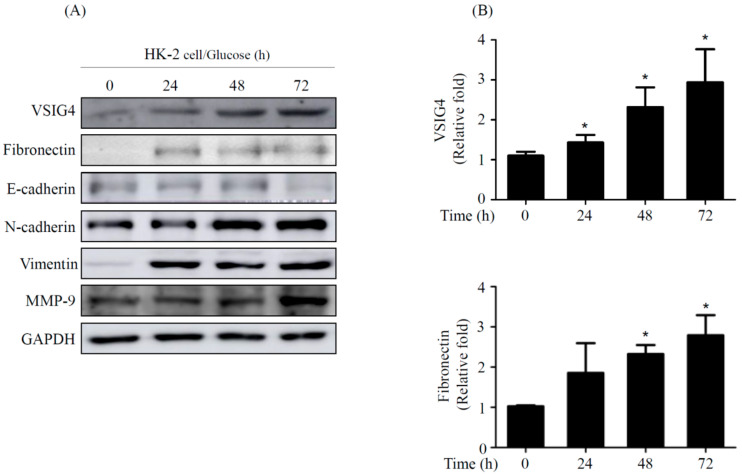
VSIG4 upregulation and changes in EMT markers in HK-2 cells grown under HG conditions. (**A**) Representative western blotting on fibrosis markers induced by HG (**B**) Expression analysis of VSIG4 and fibronectin protein using western blotting analysis. Abbreviations: HG, high glucose; EMT, epithelial-mesenchymal transition; MMP-9, matrix metalloproteinase-9. Values are expressed as mean ± SE. * *p* < 0.05 vs. LG.

As VSIG4 overexpression is known to induce EMT in renal tubular cells [9], we next investigated the protein expression of E-cadherin, N-cadherin, vimentin, fibronectin, and matrix metalloproteinase-9 (MMP-9) in HK-2 cells under HG conditions. The HG stimulation increased the protein expression of VSIG4 accompanied by moderation in the expression of fibronectin, and significant increase in the expression of N-cadherin, vimentin, and MMP-9. In contrast, the expression of the epithelial marker, E-cadherin, was reduced by HG stimulation in a time-dependent manner (Figure 2).

### 2.3. Effect of VSIG4 on EMT and Proliferation of Renal Epithelial MDCK Cells

**Figure 3:**
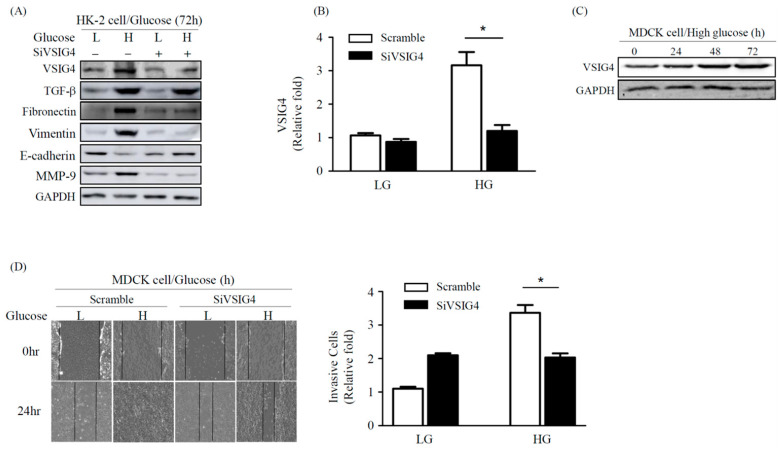
Effect of VSIG4 inhibition on EMT markers and cell motility. (**A**) HK-2 cells were transfected with siVSIG4 under HG condition for 72 h, and then cells were collected. The expression of VSIG4 and fibrosis marker was analyzed using Western blotting. (**B**) Expression of VSIG4 after siVSIG4 transfection. (**C**) Representative Madin Darby Canine Kidney (MDCK) cells were incubated under HG condition for 24 h, 48 h, and 72 h, and then subjected to Western blot analysis using an anti-VSIG4 antibody. (**D**) Cell motility assay after siVSIG4 transfection was performed and MDCK cells were photographed using a CELENA^TM^ digital imaging system. HG, high glucose; LG, low glucose; EMT, epithelial-mesenchymal transition; TGF-β, transforming growth factor-β; MMP-9, matrix metalloproteinase-9. Values are expressed as mean ± SE. * *p* < 0.01 vs. LG.

To examine the impact of VSIG4 on fibrosis in HK-2 cells stimulated by HG, the cells were transfected with VSIG4-siRNA to inhibit the activity of VSIG4. After downregulation of VSIG4 using siRNA, the increased expression of fibronectin, vimentin, and MMP-9 in HK-2 cells under HG conditions was attenuated. Furthermore, the reduced E-cadherin expression was restored after the VSIG4-siRNA transfection. Interestingly, TGF-β expression was not affected by HG stimulation after VSIG4 inhibition (Figure 3A,B).

The VSIG4 expression in Madin Darby Canine Kidney (MDCK) cells exhibited patterns similar to that observed in the HK-2 cells (Figure 3C). We then examined HG-induced cell motility using an MDCK cell system. HG was found to induce cell motility and invasion compared to low glucose (LG). These upregulations were inhibited after transfection with VSIG4-siRNA, while no changes were observed after transfection with scrambled siRNA (Figure 3D).

### 2.4. Effect of TGF-β on Fibrosis Marker in HG Conditions in HK-2 Cells

**Figure 4:**
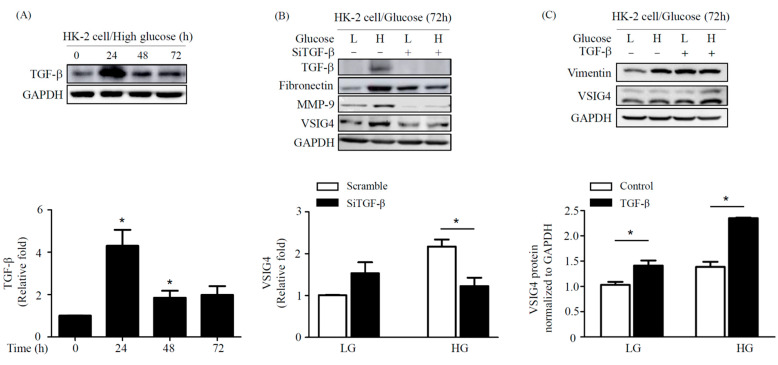
Relation between TGF-β and VSIG4 in HK cells stimulated by HG. (**A**) HK-2 cells were incubated under HG condition for 6 h, 24 h, and 72 h, and then cells were collected. The TGF-β expression was anaylzed using western blot assay. (**B**) HK-2 cells were transfected with si-TGF-β under HG condition for 72 h, and then cells were collected. (**C**) HK-2 cells were stimulated with recombinant TGF-β (10 ng/mL). The expression levels of VSIG4 and fibrosis marker was tested by Western blot assay. HG, high glucose. (**A**) * *p* < 0.05 vs. LG, (**B**,**C**) * *p* < 0.01 vs. LG

We next evaluated the association of TGF-β and VSIG4 in kidney fibrosis induced by HG. The expression of TGF-β protein in the HK-2 cells was highest at 24 h post-HG stimulation. Notably, the transfection of siTGF-β attenuated the expression of VSIG4 as well as that of fibronectin and MMP-9 in HK-2 cells induced by HG treatment (Figure 4B). In contrast, recombinant TGF-β upregulated VSIG4 expression, subsequently stimulating fibronectin expression (Figure 4C).

## 3. Discussion

This study demonstrated that HG induced VSIG4 expression, and this upregulated VSIG4 played a role in the process of EMT in renal tubular cells. Furthermore, VSIG4 was found to be a downstream mediator of TGF-β and an upstream regulator of pro-fibrotic markers. To the best of our knowledge, this is the first report about the role of VSIG4, especially in relation with TGF-β, in diabetic tubular injury.

Recently, the upregulation of VSIG4 was reported in kidney diseases, including diabetic kidney disease [10], lupus nephritis [11], and immunoglobulin A nephropathy [12]. A previous study has also suggested that VSIG4 plays an important role in the progression of EMT in kidney tubular epithelial cells [9]. Here, we demonstrated that HG upregulated VSIG4 expression accompanied by the increased expression of mesenchymal markers and decreased expression of an epithelial marker associated with EMT. Conversely, the inhibition of VSIG4 ameliorated the HG-induced abnormal expression related to the EMT of tubular epithelial cells. These results suggest that VSIG4 is involved in the HG-induced EMT in renal tubular cells.

Although the molecule critical to the EMT of tubular epithelial cells in the progression of renal tissue injury under high glucose conditions remains unknown, the multifactorial role of TGF-β signaling might have an important fibrogenic effect [4]. Acquisition of mesenchymal markers, loss of E-cadherin, and increased expression of fibronectin mediated by TGF-β are regarded as other important changes during EMT [13]. MMP-9 also regulates TGF-β-induced tubular EMT in murine renal cells [14]. Thus, we hypothesized that VSIG4 is a mediator of EMT under high glucose and it functions through regulation of TGF-β in tubular epithelial cells. In this study, the inhibition of TGF-β suppressed VSIG4 expression. However, VSIG4 inhibition suppressed the EMT pathway in renal tubular cells stimulated by HG, even though it did not reduce TGF-β expression. Therefore, VSIG4 might be a downstream mediator of TGF-β in the process of EMT of the renal tubular cells in the diabetic milieu.

In conclusion, HG conditions could upregulate VSIG4 expression and promote EMT progression. Furthermore, the inhibition of VSIG4 attenuated the HG-induced EMT progression. Considering that TGF-β is a key mediator of fibrosis in diabetic nephropathy, VSIG4 could be an important factor in the progression of diabetic nephropathy. Future studies should be considered to reveal the specific mechanism of VSIG4 in diabetic nephropathy in vivo.

## 4. Materials and Methods

### 4.1. Cells Culture and Reagents

HK-2 cells, human kidney proximal tubular epithelial cells, and MDCK cells, Madin Darby Canine Kidney cells, were obtained from the American Type Culture Collection (ATCC, Manassas, VA, USA). Both cells were cultured in keratinocyte serum-free medium (Invitrogen, Carlsbad, CA, USA) supplemented with 5 ng/mL epidermal growth factor and 0.05 mg/mL bovine pituitary extract or Dulbecco’s modified Eagle’s medium (Mediatech Inc., Corning Subsidiary, Manassas, VA, USA) supplemented with 10% heat-inactivated fetal bovine serum (Tissue Culture Biologicals, Tulare, CA, USA), 100 μg/mL streptomycin, and 100 IU/mL penicillin at 37 °C in a 5% CO_2_ humidified air condition, respectively. Cells were incubated with 5.5 mM D-glucose (low-glucose, LG) or 55 mM D-glucose (high-glucose, HG) for the indicated period (24, 48, 72 h). To confirm the relationship between TGF-β and VSIG4, cells were stimulated using 10 ng/mL recombinant TGF-β (R&D systems, Minneapolis, MN, USA).

### 4.2. RNA Interference

Cells were incubated at 5 × 10^5^ cells in 60 mm^3^ dishes, and then transfected with scrambled siRNA (Genolution Pharmaceutics, Seoul, Korea), VSIG4-siRNA (z391g siRNA), or TGF-β-siRNA (Santa Cruz Biotechnology, Santa Cruz, CA, USA) for 72 h using Lipofectamine RNAi Max (Invitrogen, Carlsbad, CA, USA) according to the manufacturer’s protocol.

### 4.3. Western Blot Analysis

Cell were lysed with radioimmunoprecipitation lysis buffer (0.1% NP-40, 50 mM 4-(2-hydroxyethyl)-1-piperazineethanesulfonic acid (HEPES) pH 7.4, 1 mM ethylenediaminetetraacetic acid (EDTA), 2.5 mM ethylene glycol-bis (β-aminoethyl ether)-N,N,N′,N′-tetraacetic acid (EGTA), 150 mM NaCl, 1 mM Dithiothreitol (DTT) including protease inhibitor cocktail (Sigma-Aldrich, St. Louis, MO, USA). A Bradford assay kit (Bio-Rad, Hercules, CA, USA) was used to measure protein concentrations. Approximately 20–30 μg of cell lysates was subjected to 10–12% SDS-PAGE. Separated proteins were transferred to Immobilion NC transfer membrane (EMD Millipore, Billerica, MA, USA). Transferred membranes were blocked using 5% skim milk in tris-buffered saline with 0.1% Tween ® 20 Detergent (TBST) buffer (0.1% Tween 20, 150 mM NaCl, 20 mM Tris-HCl, pH7.4) and then incubated with the following primary antibodies at 4 °C overnight: anti-VSIG4, anti-E-cadherin, anti-N-cadherin (Abcam, Cambridge, MA, USA), anti-Vimentin, anti-TGF-β, anti-fibronectin, anti-matrix metalloproteinase-9 (MMP-9) (Cell Signaling Technology, Beverly, MA, USA), and anti- glyceraldehyde 3-phosphate dehydrogenase (GAPDH) (Aviva Systems Biology, San Diego, CA, USA). The primary antibodies were reacted with horseradish peroxidase-conjugated anti-mouse or anti-rabbit secondary antibodies (Cell Signaling Technology, Beverly, MA, USA) and enhanced using Enhanced chemiluminescence (ECL) detection reagents (Amersham, Buckinghamshire, UK). The blot images were recorded using AI600 (GE Healthcare Bio-Sciences Corp., Marlborough, MA, USA).

### 4.4. Reverse Transcription Polymerase Chain Reaction (RT-PCR) Analysis

Collected cell pellets were incubated with 1 mL Trizol reagent (Thermo Fisher Scientific Inc., Waltham, MA, USA). Isolated total RNA was reverse transcribed to cDNA using an AccuPower cycle RT premix kit (Bioneer, Daejeon, Korea). To validate the expression of VSIG4, PCR amplification was performed using following specific primers: VSIG4 (forward: 5′-AACTCTCTGTCTCCAAGCCC-3′; reverse: 5′-GCAGTGCAGAAATAGGAGCC-3′), and β-actin (forward: 5′-GCCGGGACCTGACTGACTAC-3′; reverse: 5′-TCTTCTCCAGGGAGGAGCTG-3′).

### 4.5. Cell Motility and Invasion Assay

MDCK cells were seeded in 6-well plates (10^5^ cells per well) and then incubated for 24 h in serum-free medium. MDCK cells were transfected with scrambled RNA or siRNA for VSIG4 for 24 h, and then the cells were seeded in 6-well plates. Cells were observed after crystal violet staining and photographed using a CELENA^TM^ Digital Imaging System.

### 4.6. Statistical Analysis

All experiments were observed independently at least three times. Data were represented as means ± SE. Statistical analysis was performed using GraphPad Prism version 5 for Windows (GraphPad Software, San Diego, CA, USA). The expression levels were analyzed a two-tailed Student’s *t*-test for quantitative variables. A *p*-value < 0.05 was regarded as statistically significant.

## Abbreviations

HG	high glucose
EMT	epithelial-mesenchymal transition
VSIG4	V-set immunoglobulin-domain-containing 4
TGF	transforming growth factor
MDCK	Madin Darby Canine Kidney
